# Affine transform representation for reducing calibration cost on absorption-based LWIR depth sensing

**DOI:** 10.1038/s41598-024-77612-2

**Published:** 2024-11-02

**Authors:** Takahiro Kushida, Ryutaro Nakamura, Hiroaki Matsuda, Wenhao Chen, Kenichiro Tanaka

**Affiliations:** College of Information Science and Engineering, Ritsumeikan University, 2-150 Iwakura-cho, Ibaraki, Osaka 567-8570 Japan

**Keywords:** Electrical and electronic engineering, Computer science

## Abstract

Multispectral long-wave infrared (LWIR) ranging is a technique that estimates the distance to the object based on wavelength-dependent absorption of LWIR light through the air. Prior works require time-consuming measurements for calibration and solve non-linear inverse problems, which sometimes falls into a local minimum. In this paper, we propose a linear representation that connects the measurements and the scene parameters using the affine matrix. In this representation, the distance and the temperature of the object can be obtained as a closed-form solution and the calibration cost can be reduced to at least three observations. In real-world experiments, we demonstrate that our method is effective to reduce the calibration cost while keeping the precision of the depth estimation.

## Introduction

Depth sensing technology is an important research topic in computer vision, which has a wide variety of applications including robotics, augmented reality, autonomous vehicles, and 3D reconstruction. Various depth sensing techniques including stereo vision, depth from focus/defocus, time-of-flight, structured light, and photometric stereo have been developed to meet the diverse needs of these applications^1^.

While these methods are effective, they have different pros and cons, and there are still some challenges left. One of the challenging scenarios is to measure the scene depth without any controlled light sources, where the scene is texture-less or under dark environments. Recently, a few passive depth sensing methods that work in such a scenario are proposed^2,3^. These works utilize the atmospheric absorption of ambient long-wave infrared (LWIR) light to estimate the depth, temperature, and emissivity from three bandwidth observations^2^ or hyperspectral observations^3^.

Although these works have demonstrated the feasibility of absorption-based LWIR depth sensing, they require a high cost calibration process. These methods assume known absorption coefficients and camera’s response function and the estimation process of these parameters requires a lot of beforehand observations by putting a blackbody furnace at different depth and temperature, which is time-consuming. Besides, these parameters must be precisely calibrated to avoid the local minima as the solution of non-linear inverse problems.

In this paper, we propose an affine transform representation by introducing Wien’s approximation. We show that an affine matrix linearly connects observations and the scene parameters, i.e., depth and temperature. In this representation, the solution can be analytically obtained and the calibration process is turned into obtaining an affine matrix instead of estimating the precise coefficients. Analogous to the affine matrix estimation in traditional image processing, the calibration cost can be reduced to at least three observations. We also extend this calibration process to include the response function of the multispectral LWIR camera. Finally, the data required for the calibration can be reduced to four observations. In the experiments, we demonstrate that our proposed method achieves the same level of accuracy as the previous methods despite much fewer measurements.

*Scope:* This work’s purpose is to reduce the number of measurements for the calibration. While the precision of the depth measurement is competitive to the prior work, the precision is not the claim of this paper.

## Related work

We briefly review the related work from two perspectives: passive depth sensing and spectral analysis in LWIR.

### Passive depth sensing

Passive depth sensing is a fundamental challenge that has been studied for a long time^1^. Various methods have been proposed to estimate depth from images and sensors in challenging conditions, such as low-light or dark environments.

Traditional stereo camera setups and multi-view stereo are widely used for depth estimation^4,5^. These methods use two or more images of a scene from different viewpoints to estimate depth through geometric and photometric consistency across the images. However, these methods face limitations in dark scenes where the visibility of features and texture is greatly reduced^6^. The lack of well-defined image features makes it difficult for these techniques to estimate depth accurately.

Another conventional approach for depth estimation is depth from focus/defocus, which analyzes the amount of blur in images^7,8^ In dark scenes, where visibility and illumination are limited, capturing images with a sufficient amount of blur variation becomes challenging.

To address the challenges posed by dark scenes, the use of thermal (LWIR) cameras for depth sensing has been explored^6,9–11^. Thermal cameras capture thermal light emitted by objects, which is independent of visible light conditions^12^. They can work effectively in darkness, offering a potential solution to the limitations of traditional stereo and focus-based methods. However, these methods still rely on object textures, and they also face challenges in long-range depth estimation due to the lack of detailed disparity information.

Recently, absorption-based depth sensing using an LWIR camera that works in dark scenes and is capable of long-range measurements has been proposed^2,3,13–15^. These approaches exploit the atmospheric absorption of the LWIR light from the environment and estimates the depth, temperature and emissivity by non-linear optimization using either three wavelength images^2^ or hyperspectral images^3,13–15^. These methods rely on the wavelength-dependent atmospheric absorption characterized by an extinction coefficient, and the calibration for estimating the coefficient requires a lot of observations. Our proposed method is to improve absorption-based depth sensing using multi-spectral LWIR images by reducing the cost of calibration and estimating the depth without non-linear optimization. Our method provides a close-form solution from multi-spectral thermal images to the scene depth. While the other methods^2,3,13–15^ use either numerical optimization or neural network to estimate the scene depth, our method only compute the matrix multiplication, which obviously reduces computational complexity.

### Spectral analysis in LWIR

While common LWIR cameras use all LWIR ranges (8–14 μm), spectral analysis is useful for various applications. Gases have unique emission and absorption spectra in LWIR region depending on their types. Spectral observations are used to detect and identify the gases^16–21^. In the field of remote sensing, LWIR spectral features are widely used for the analysis of soil^22,23^, mineral^23–25^, and the estimation of vegetation^23,26^. For a comprehensive overview of spectral analysis in LWIR, we refer the reader to the survey of LWIR hyperspectral imaging^27^. We also utilize the multispectral LWIR observations to a new application that jointly estimates the scene depth and its temperature.

## Passive depth sensing with multi-spectral LWIR measurements

We briefly review the prior work^2^ and discuss the remaining issues. According to Planck’s law, the spectral radiance of a black body $M_e$ for wavelength $\lambda$ at absolute temperature $T$ is given by

1 $$\begin{aligned} M_e(\lambda ; T) = \frac{2\pi hc^2}{\lambda ^5} \frac{1}{e^{hc/ \lambda kT}-1}, \end{aligned}$$

where $h$ is the Planck constant, $k$ is the Boltzmann constant, and $c$ is the speed of light. Real objects are not ideal black bodies and the spectral radiance $E$ from objects is represented as

2 $$\begin{aligned} E(\lambda ; \epsilon , T) = \epsilon M_e(\lambda ;T), \end{aligned}$$

where $\epsilon$ is emissivity, which is the ratio to the radiance of a black body and depends on the material and surface structure.

The air absorbs the light emitted from objects while the light travels from the object to the camera. Lambert Beer’s law gives the amount of attenuation with the traveling distance $d$^28^:

3 $$\begin{aligned} i_{\text {out}}(\lambda ) = \exp {(-\sigma (\lambda )d)} i_{\text {in}}(\lambda ), \end{aligned}$$

where $i_{\text {out}}$ and $i_{\text {in}}$ denote the intensity after and before attenuation, respectively, and $\sigma$ denotes the extinction coefficient of the air. Using Eqs. (1)–(3), we can express the observation model as:

4 $$\begin{aligned} I(\lambda ) = R_v(\lambda )\exp (-\sigma (\lambda )d)\epsilon M_e(\lambda ; T), \end{aligned}$$

where $R_v$ is the camera’s sensitivity.

Equation (4) has three unknown parameters $d, T$ and $\epsilon$. Previous methods^2,3^ show that these unknowns can be estimated with more than three observations at different wavelengths. However, these methods have two common problems. First, they require a large number of measurements for calibration. The extinction coefficient $\sigma$ and camera sensitivity $R_v$ should be calibrated beforehand, which requires many measurements as these parameters are wavelength-dependent. Second, solving the inverse problem of this equations is a non-linear optimization so estimating $d$ and $T$ is computationally expensive and prone to local minima, i.e., the solution is sensitive to the coefficients or the observations. To stably calibrate the coefficients, the measurements for the calibration tend to increase.

## Proposed method

In this paper, we propose a linear approximation representation that reduces the number of observations for calibration to at least three measurements and enables the analytical solution of $d$ and $T$ by solving a linear system of equations.

### Linear approximation by Wien’s distribution law

 Black body radiation can be approximated by Wien’s distribution law when $hc/\lambda \gg kT$ in the region of the short wavelength spectrum of thermal radiation^28^. According to the Wien’s approximation, black body radiation can be represented by,

5 $$\begin{aligned} M_e(\lambda , T) = \frac{2\pi ch^2}{\lambda ^5}\exp \left( {-\frac{hc}{\lambda kT}}\right) . \end{aligned}$$

By taking the ratio of two observations at two different wavelengths $I(\lambda _i)$, $I(\lambda _j)$ and using Eqs. (4) and (5), we get

6 $$\begin{aligned} \frac{I(\lambda _i)}{I(\lambda _j)} =&\frac{R_v(\lambda _i)\lambda _j^5}{R_v(\lambda _j)\lambda _i^5} \exp \left( -(\sigma (\lambda _j)-\sigma (\lambda _i))d\right) \exp \left( -\frac{hc}{(\lambda _i-\lambda _j)k}\frac{1}{T}\right) . \end{aligned}$$

Taking the logarithm of both sides, we get

7 $$\begin{aligned} I_{i, j} = \log \left( \frac{I(\lambda _i)}{I(\lambda _j)}\right)&= C^{i,j}_1 + C^{i,j}_2d + C^{i,j}_3T', \end{aligned}$$

where $T' = \frac{1}{T}$ is the inverse temperature and the constants are given by

8 $$\begin{aligned} C^{i,j}_1&= \log \left( \frac{R_v(\lambda _i)\lambda _j^5}{R_v(\lambda _j)\lambda _i^5}\right) , \end{aligned}$$

9 $$\begin{aligned} C^{i,j}_2&= -\left( \sigma (\lambda _j)-\sigma (\lambda _i)\right) , \end{aligned}$$

10 $$\begin{aligned} C^{i,j}_3&= -\frac{hc}{(\lambda _i-\lambda _j)k}. \end{aligned}$$

Apparently, the logarithm of the ratio of measurements $I_{i, j}$ varies linearly with respect to the distance $d$ and the inverse of temperature $T'$. Using measurements at three different wavelengths $\lambda _i, \lambda _j$ and $\lambda _l$, two sets of measurement pairs are obtained as follows:

11 $$\begin{aligned} I_{i, j}&= C^{i,j}_1 + C^{i,j}_2d + C^{i,j}_3T', \end{aligned}$$

12 $$\begin{aligned} I_{l, j}&= C^{l,j}_1 + C^{l,j}_2d + C^{l,j}_3T'. \end{aligned}$$

Denoting the matrix as

13 $$\begin{aligned} \textbf{A} = \begin{bmatrix} C^{i,j}_2 & C^{i,j}_3 & C^{i,j}_1 \\ C^{l,j}_2 & C^{l,j}_3 & C^{l,j}_1 \\ 0 & 0 & 1 \end{bmatrix}, \end{aligned}$$

we obtain

14 $$\begin{aligned} \begin{bmatrix} I_{i,j} \\ I_{l,j} \\ 1 \\ \end{bmatrix} = \textbf{A} \begin{bmatrix} d \\ T' \\ 1 \end{bmatrix} . \end{aligned}$$

**Fig. 1:**
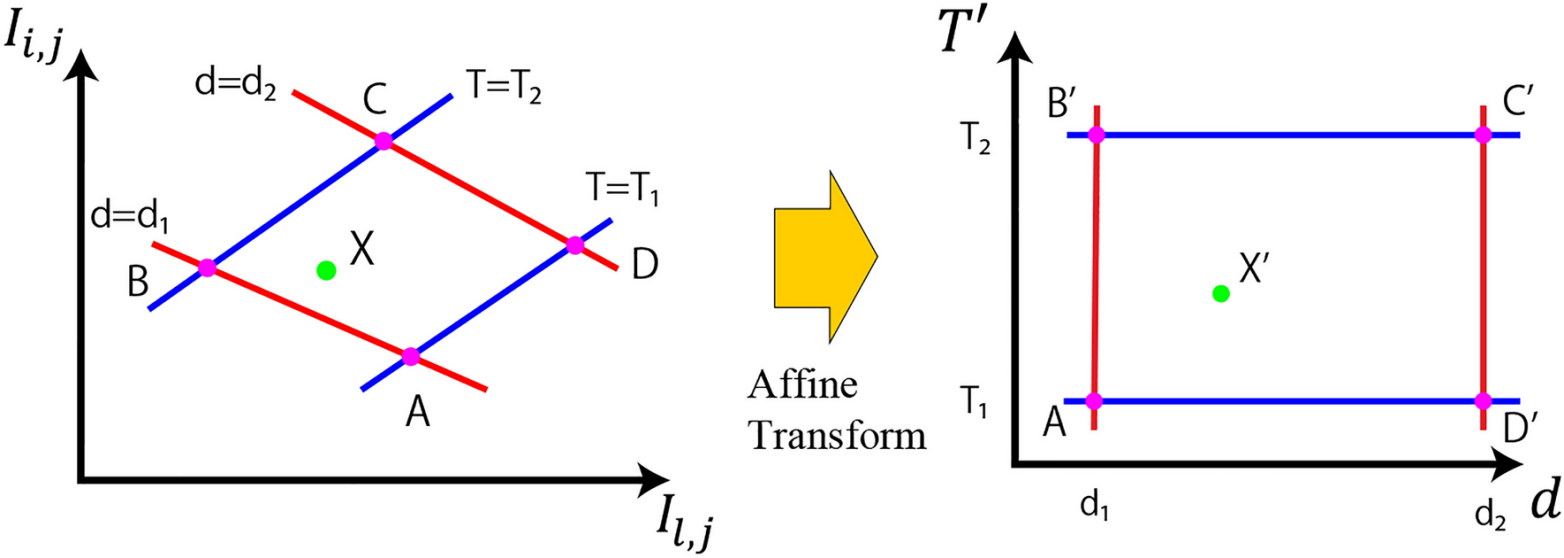
The relationship between the pairs of observations $(I_{i, j}, I_{l, j})$ and the pair of distance and temperature $(d, T')$ can be expressed by an affine transformation. We estimate the distance and temperature $(d, T')$ from the observations $(I_{i, j}, I_{l, j})$ using the affine transformation. The affine transformation matrix can be obtained from at least three corresponding points. In this study, we perform the end-to-end calibration of the entire observation system using four corresponding points.

As the matrix $\textbf{A}$ has the form of affine transformation^1^, each of its elements can be estimated from at least three observations of the object at different distances and temperatures analogous to the traditional image processing. Once the matrix is obtained, the distance $d$ and the inverse of temperature $T'$ can be obtained by multiplying the inverse of $\textbf{A}$ as shown in Fig. 1.

### Extension to photometric calibration of observation system

**Fig. 2:**
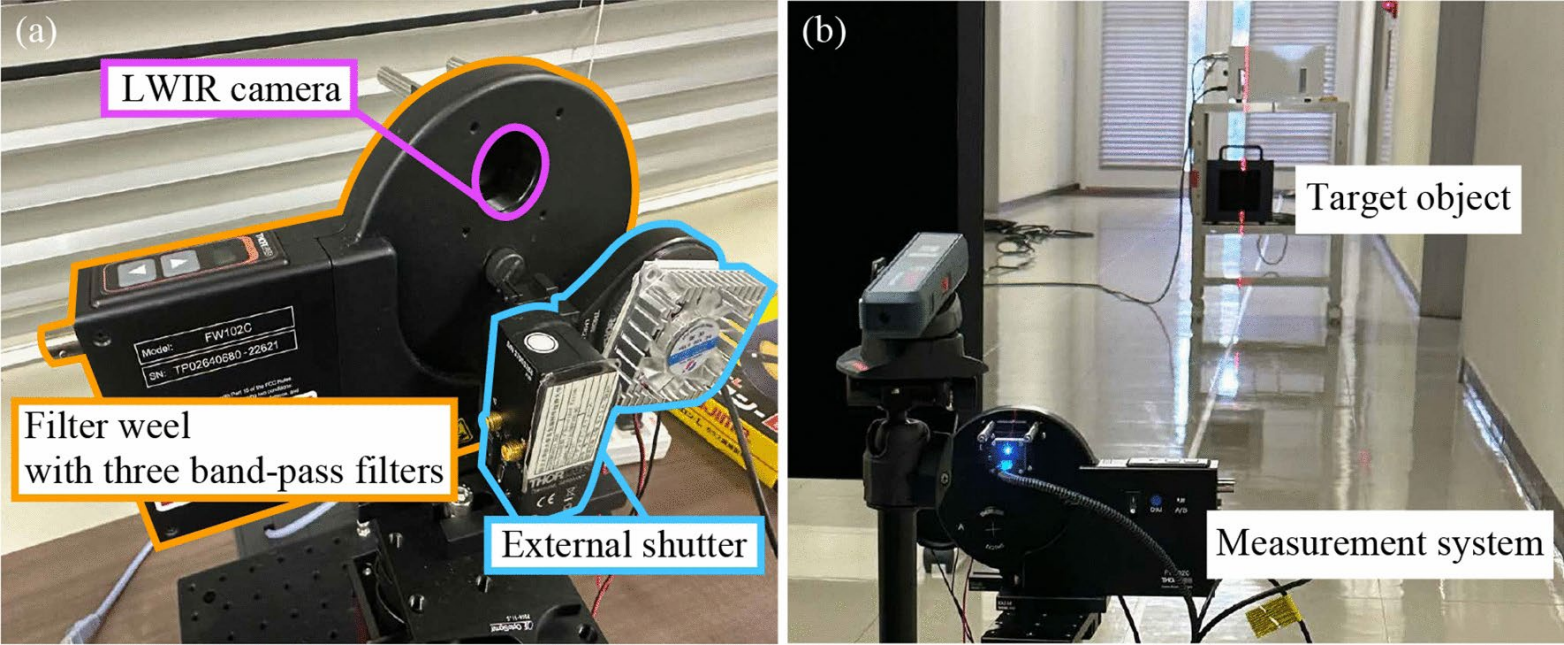
Experimental setup. (**a**) We build a multi-spectral LWIR measurement system. The system consists of a LWIR camera, a filter wheel with three band-pass filters, and an external shutter. (**b**) We capture a black body furnace for a target object. The black body furnace and the camera are placed in a straight line, and images are captured for each temperature and distance.

 In our implementation, we further add an extra measurement to include the calibration of the response function of the multispectral LWIR camera. In multispectral LWIR systems including the prior work^2^ and ours, several bandpass filters are placed in front of the camera to get multi-spectral LWIR images. Unfortunately, this type of setup suffers from the narcissus effect, which is caused by the camera’s self-emission reflected off the filter. To mitigate this effect, an external shutter is often used as shown in Fig. 2. The observed images with the shutter opened $I_{\text{open}}(\lambda )$ and closed $I_{\text{close}}(\lambda )$ are expressed as

15 $$\begin{aligned} I_{\text{open}}(\lambda )&= I_{\text{scene}}(\lambda ) + I_{\text{ref}}(\lambda ),\end{aligned}$$

16 $$\begin{aligned} I_{\text{close}}(\lambda )&= I_{\text{ref}}(\lambda ) + I_{\text{shutter}}(\lambda ), \end{aligned}$$

respectively, where $I_{\text{scene}}(\lambda )$ is the intensity of the scene, $I_{\text{ref}}(\lambda )$ is the intensity reflected off the filter and $I_{\text{shutter}}(\lambda )$ is the intensity of the thermal radiation from the shutter. By subtracting Eq. 15 from Eq. 16 and solving for $I_{\text{scene}}(\lambda )$, we get

17 $$\begin{aligned} I_{\text{scene}}(\lambda )&= I_{\text{open}}(\lambda ) - I_{\text{close}}(\lambda ) + I_{\text{shutter}}(\lambda ) \end{aligned}$$

18 $$\begin{aligned}&= I_{\text{diff}}(\lambda ) + I_{\text{shutter}}(\lambda ), \end{aligned}$$

where we replace $I_{\text{open}}(\lambda ) - I_{\text{close}}(\lambda )$ to $I_{\text{diff}}(\lambda )$ for simplification. The previous work estimates the intensity of the thermal radiation from the shutter $I_{\text{shutter}}(\lambda )$ by measuring the temperature of the shutter directly and applying Planck’s law^2^. This approach is, however, not so accurate as the ideal model does not match the real world. In our method, we estimate the intensity $I_{\text{shutter}}(\lambda )$ based on the measurements of the calibration target as well as the affine matrix $\textbf{A}$.

The observations of the calibration target at the combination of two different temperatures $T_1, T_2$ and distances $d_1, d_2$ are expressed by Eq. 4 as

19 $$\begin{aligned} I_{\text{scene}}(\lambda ;d_1, T_1)&= R_v(\lambda )\exp (-\sigma (\lambda )d_1)\epsilon M_e(\lambda ; T_1),\end{aligned}$$

20 $$\begin{aligned} I_{\text{scene}}(\lambda ;d_1, T_2)&= R_v(\lambda )\exp (-\sigma (\lambda )d_1)\epsilon M_e(\lambda ; T_2),\end{aligned}$$

21 $$\begin{aligned} I_{\text{scene}}(\lambda ;d_2, T_1)&= R_v(\lambda )\exp (-\sigma (\lambda )d_2)\epsilon M_e(\lambda ; T_1),\end{aligned}$$

22 $$\begin{aligned} I_{\text{scene}}(\lambda ;d_2, T_2)&= R_v(\lambda )\exp (-\sigma (\lambda )d_2)\epsilon M_e(\lambda ; T_2). \end{aligned}$$

By taking the ratio of two observations at different distances but the same temperature, we get

23 $$\begin{aligned} \frac{I_{\text{scene}}(\lambda ;d_1, T_1)}{I_{\text{scene}}(\lambda ;d_2, T_1)} = \frac{I_{\text{scene}}(\lambda ;d_1, T_2)}{I_{\text{scene}}(\lambda ;d_2, T_2)} = \frac{\exp (-\sigma (\lambda )d_1)}{\exp (-\sigma (\lambda )d_2)} \end{aligned}$$

Substituting Eq. 18 into Eq. 23 and solving for $I_{\text{shutter}}(\lambda )$ yields

24 $$\begin{aligned} I_{\text{shutter}}(\lambda ) = \frac{I_{\text{diff}}(\lambda ;d_1, T_1) I_{\text{diff}}(\lambda ;d_2, T_2) - I_{\text{diff}}(\lambda ;d_2, T_1) I_{\text{diff}}(\lambda ;d_1, T_2)}{I_{\text{diff}}(\lambda ;d_2, T_1) + I_{\text{diff}}(\lambda ;d_1, T_2) - I_{\text{diff}}(\lambda ;d_1, T_1) - I_{\text{diff}}(\lambda ;d_2, T_2)}. \end{aligned}$$

Equation 24 shows that the intensity from the shutter $I_{\text{shutter}}(\lambda )$ can be obtained from four observations at different temperatures and distances. Finally, we can express all calibration parameters in the measurement domain instead of estimating physical coefficients of the system.

**Fig. 3:**
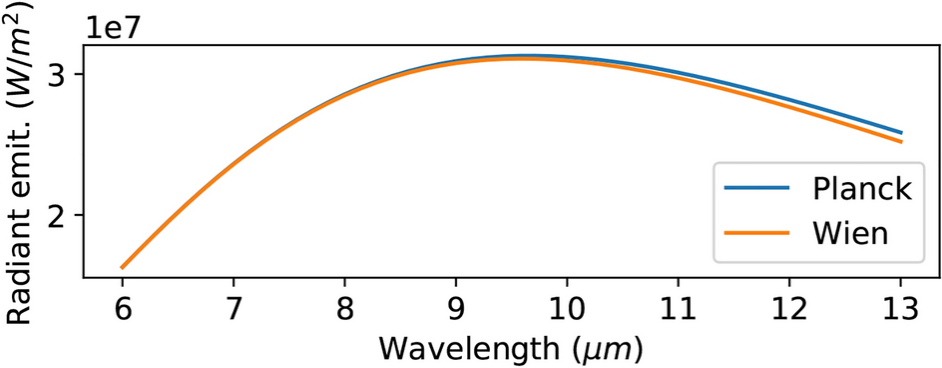
Comparison of spectral radiance of Planck’s law and Wien’s law at 300K. Wien’s law shows a similar spectral radiance in our target spectral range (8–13 μm).

## Experiments

### Validity of Wien’s approximation

Our proposed method uses Wien’s approximation to transform the non-linear observation model into a linear model. Here we discuss the validity of Wien’s approximation for black body radiation. The temperature and wavelength range of the target objects in this study are 270–370K and 8–13 μm, respectively. Figure 3 shows the spectral radiance of Plank’s law and Wien’s law at 300K. The relative error between these two models is 0.89%, indicating that Wien’s approximation provides a sufficiently accurate approximation in the target range of our study.

### Real-world experiments

#### Milti-spectral LWIR observation system

 We built a similar observation system as previous work^2^ as shown in Fig. 2a. Only Nagase *et al.*’s system^2^ is reproducible as they use off-the-shelf components while any other system^3,13–15^ is not publicly accessible, we can only compare against Nagase *et al.*.^2^ The system consists of a thermal camera (FLIR Boson 640 40 mm, $\text{NETD}={40}\hbox {mK}$), a filter wheel with three narrow bandpass filters, and an external shutter. The wavelength of each filter is $\lambda _i={8}\,{\upmu }\,\hbox {m}~(\text{CWL}~{8.248}\,{\upmu }$ m, FWHM 452 nm), $\lambda _j={9}\,{\upmu }\hbox {m}~(\hbox {CWL}~{9.127}\,{\upmu }$m, FWHM 545 μm), and $\lambda _l={10}\,{\upmu }\hbox {m}~(\hbox {CWL}~{10.400}{\upmu }$m, FWHM 737 μm), respectively. The shutter is sprayed with black body paint and cooled using a Peltier device. The shutter is used to remove the narcissus effect and reduces imaging noise.

#### Data collection

 We capture a black body furnace by changing the temperatures and distances as shown in Fig. 2b. The temperature is set to four different temperatures: 60 °C, 70 °C, 80 °C and 90 °C. The distance ranges from 1 to 15 m, increasing at 1 m intervals. We use the data of 60 °C and 90 °C for calibration and 70 °C and 80 °C for evaluation. Images at three wavelengths are captured by changing the filters for each distance and temperature.

#### Calibration

 We use the following 4 sets of temperature and distance to estimate the matrix $\textbf{A}$ in our proposed method: (1 m, 60 °C), (10 m, 60 °C), (1 m, 90 °C), (10 m, 90 °C). Regarding the previous work, the camera sensitivity $R_v$ and the extinction coefficient $\sigma$ are estimated in the calibration. For these estimations, we use all distance measurements at temperatures of 60 °C and 90 °C, 30 sets of observations in total.

#### Results

**Fig. 4:**
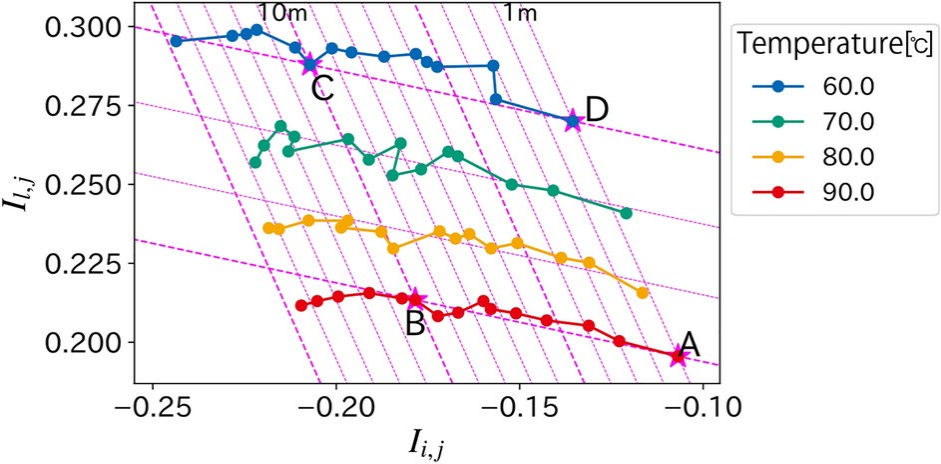
A visualization of the pair of all observations $(I_{i,j}, I_{l,j})$. The solid line represents the observations with the same temperature and the color represents the temperature. We use four points A–D to estimate the affine matrix. The magenta dotted line represents the projected points of the depth (from 1 to 15 m in steps of 1m) and temperature (from 60 to 90 °C in steps of 10 °C) using the estimated matrix.

First, we visualize the pair of all observations $(I_{i,j}, I_{l,j})$ shown in Fig. 4. The points from 1 to 15 m at each temperature are connected sequentially in the order of their decreasing distance. Points A to D are the four points used to estimate the affine matrix. The magenta dotted line represents the projection of points where the depth $d$ ranges from 1 to 15 m, and the temperature $T$ of 60 °C, 70 °C, 80 °C and 90 °C. Each point is regularly aligned and matches the magenta grid. The results show that our proposed method can estimate the depth and temperature using an affine transformation matrix estimated using only four-point observations.

**Fig. 5:**
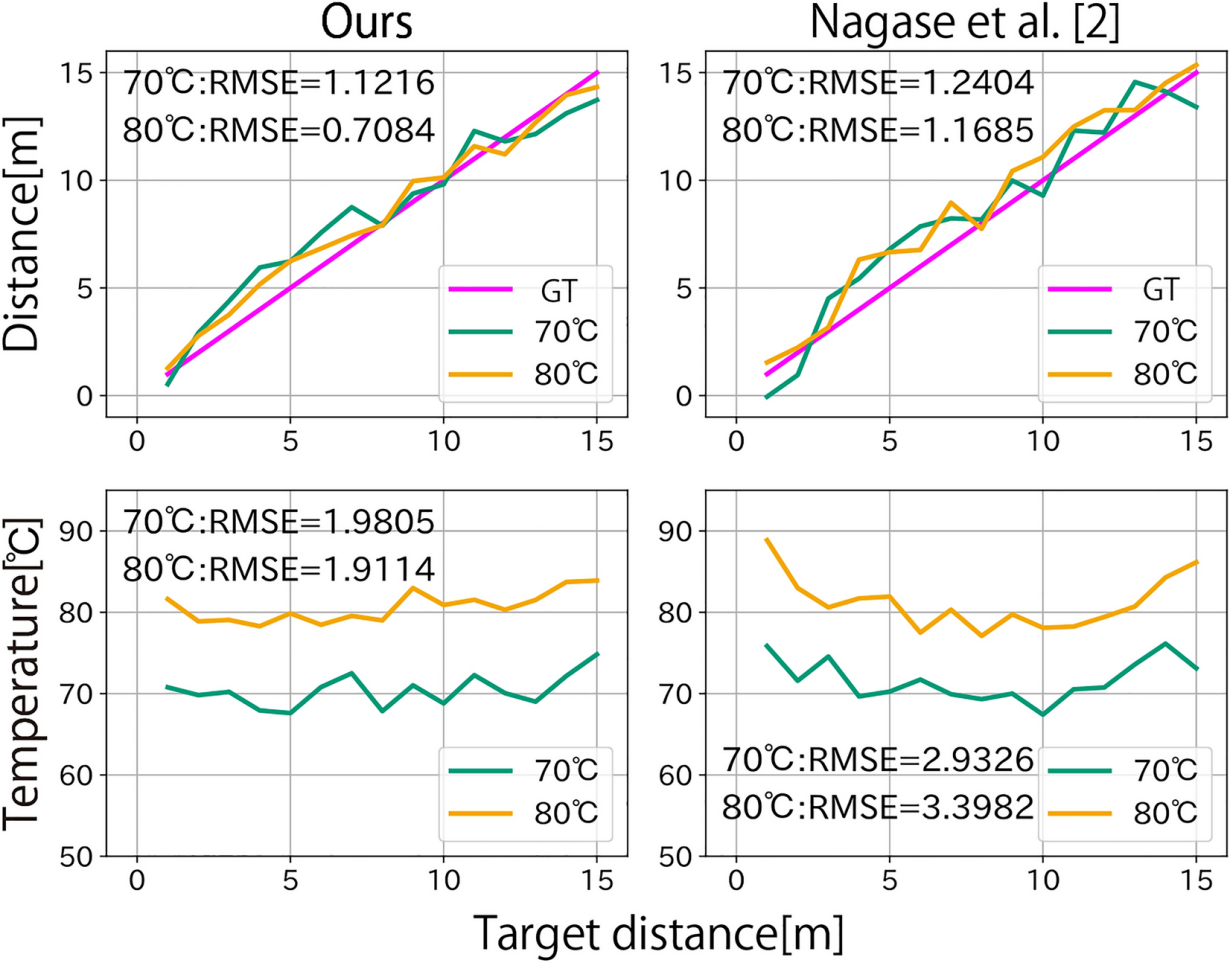
Comparison of the estimated depth and temperature. Our proposed archives a similar accuracy with the previous work^2^ while the amount of data for calibration is reduced.

Next, we compare the results of estimated distance and temperature using our method with those of the previous method^2^ shown in Fig. 5. The green and orange lines are the estimated results from the observations at 70 °C and 80 °C using the affine matrix obtained by four points A–D in Fig. 4, respectively. We use root mean square error (RMSE) between the ground truth and estimated results for quantitative evaluation. Although our method uses significantly less data for calibration (4 vs. 30), both distances and temperatures are estimated accurately as RMSE is at the same level.

**Fig. 6:**
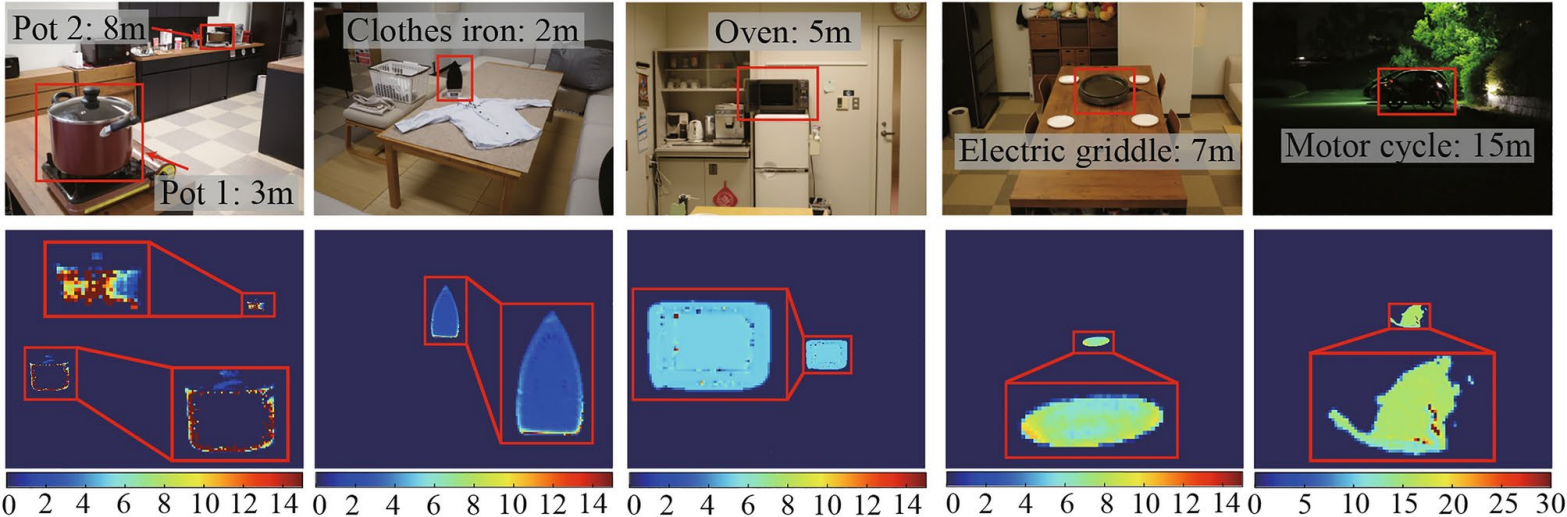
Demonstration on real scenes. The upper row shows the scene images and the targets. Target distances are roughly measured using a scale and the room light is turned off while the measurement. The bottom row shows the estimated results. The background is manually masked out. It is shown that the depth of the target can be measured in dark scenes.

### Demonstration

For a demonstration of our method, we apply the algorithm to entire the image. Figure 6 shows the qualitative results on real-world scenes including dark environment. The target objects are a pot, a clothes iron, an oven, a griddle, and a motorbike as shown in the upper row of Fig. 6. The distances of the objects are roughly measured by a scale. The calibration process is carried out using the black body furnace. We use the same filters as Section 5.2 and the depth is estimated pixel by pixel using the same algorithm. The background of the image is masked out in the same way of the previous work^2^.

The estimated scene depth is shown in the bottom row of Fig. 6. It is shown that the depths of the target objects are correctly estimated. Especially in the motorbike scene, it is shown that our method works well in dark environment, where other passive approaches including stereo, depth from focus/defocus, and shape from shading are not applicable.

## Conclusion

This paper presents a simplified approach for absorption-based multi-wavelength LWIR ranging that avoids the time-consuming calibration and non-linear optimization. By applying Wien’s approximation to Planck’s thermal radiation law, the observation model is linearized and the relationship between the measured intensity and the scene parameters of temperature and distance is expressed by an affine transformation. The inverse problem can be solved analytically by estimating the affine matrix from only four measurements. Experimental results in a real-world environment show that the method can estimate both temperature and distance without loss of accuracy compared to existing methods.

In future work, we are interested in exploring the calibration method using fewer images or improving the accuracy using learning-based approaches.

## Data Availability

The datasets analyzed during the current study are available from the corresponding author upon reasonable request.
